# Effect of Upper Arm Position Changes on the Occurrence of Ipsilateral Shoulder Pain After Single-Operator Port Thoracoscopy

**DOI:** 10.3389/fsurg.2022.823259

**Published:** 2022-02-02

**Authors:** Dian Ren, Bo Zhang, Jie Xu, Renwang Liu, Jing Wang, Huandong Huo, Hao Zhang, Jingtong Zeng, Hanqing Wang, Xiaohong Xu, Mei Lin, Song Xu, Zuoqing Song

**Affiliations:** ^1^Department of Lung Cancer Surgery, Lung Cancer Institute, Tianjin Medical University General Hospital, Tianjin, China; ^2^Colleges of Nursing, Tianjin Medical University, Tianjin, China; ^3^Tianjin Key Laboratory of Lung Cancer Metastasis and Tumour Microenvironment, Lung Cancer Institute, Tianjin Medical University General Hospital, Tianjin, China; ^4^Department of Nursing, Tianjin Medical University General Hospital, Tianjin, China

**Keywords:** ISP, arm position, surgical trauma, E-RAS, nursing

## Abstract

**Background:**

The aim of this study was to explore the factors associated with the occurrence of ISP after VATS to reduce the incidence of ISP and improve patients' quality of life.

**Methods:**

The data of patients were collected between June 2020 and August 2020 in the Department of Lung Cancer Surgery, Tianjin Medical University General Hospital. The angle of upper arm was measured intraoperatively. The patient's postoperative shoulder function was quantified using the Constant-Murley shoulder function rating score. The proportional hazards model was applied to identify multiple influence factors.

**Results:**

A total of 140 eligible patients met criteria. At postoperative day 3, only the age influenced patients' shoulder pain. At postoperative day 14, univariate and multivariate logistic regression analyses showed that age (odds ratio [OR]: 1.098 [1.046-1.152]; *P* < 0.001) and upper arm Angle A (OR: 1.064 [1.011-1.121]; *P* = 0.018) were independent risk factors for low shoulder function scores. However, height was its protective factor (OR: 0.923 [0.871-0.977]; *P* = 0.006). At postoperative day 42, univariate and multivariate logistic regression analyses showed that age (OR: 1.079 [1.036-1.124]; *P* < 0.001) was a risk factor for low shoulder function scores, and height (OR: 0.933 [0.886-0.983]; *P* = 0.009) was its protective factor. In contrast, upper arm Angle B was not statistically associated with low shoulder function scores (P>0.05). In addition, the reduction in ipsilateral Shoulder scores after surgery was higher in patients with more than 113° of angle A (*P* = 0.025).

**Conclusion:**

ISP was closely related to the angle of anterior flexion of the upper arm on the patient's operative side intraoperatively. The increase in the degree of postoperative shoulder injury is more pronounced for an anterior flexion angle of >113°. Therefore, we recommend that the angle of anterior flexion of the upper extremity should be <113° intraoperatively.

## Introduction

Single-operator port thoracoscopic surgery is technically well established and accounts for more than 80% of thoracic surgical procedures. Therefore, thoracic surgeons are now more dedicated to explore the rapid recovery of single-operator port thoracoscopic surgery to maximize the advantages of minimally invasive surgery.

A proportion of patients experience postoperative shoulder pain after single-operator port thoracoscopic surgery, known as ipsilateral shoulder pain (ISP), which was first defined by Mark and Brodsky (1, 2). The causes of ISP are multifaceted, including surgical damage to muscles and nerves (3–6), and we found that the occurrence of ISP also correlated with intraoperative upper limb position. Although most patients' shoulder pain can be relieved in the postoperative period (7), exploring ways to minimize this medically induced injury from the perspective of operating room management and promoting the patient's rapid postoperative recovery is necessary.

The incidence of ISP varies significantly owning to the volume of surgery, difficulty of surgery, and ratio of conventional open thoracic surgery to video-assisted thoracoscopic surgery (VATS) surgery in different medical centers. ISP is most likely to occur within 4 days postoperatively, after which shoulder pain gradually subsides (7). Furthermore, shoulder pain occurring during this time is triggered by intraoperative manipulation that damages the nerves and muscles; hence, the symptoms disappear quickly. Single-operating port thoracoscopic surgery is relatively less damaging to the nerves and muscles, and it triggers ISP largely because of periapical muscle ligament injury, which has a long recovery time and can last until 1 month after surgery (6). A few patients can experience chronic pain 1 year postoperatively (8). The incidence of ISP in thoracic surgery ranges between 21 and 97% (4, 9, 10) and recent literature indicates a prevalence rate of 42–85% (11). The incidence of ISP after single-operator port thoracoscopy is ~20%, which is significantly lower than the incidence of ISP after open thoracic surgery (10).

The causes of ISP are still inconclusive, but are broadly divided into two categories, namely neurological and physical, with neurological causes being mainly phrenic nerve pain (3–5) and visceral pain caused by the visceral pleura and pericardium (5), and physical causes being mainly periapical muscle ligament injuries (6, 12), in addition to main bronchial transection (1) and pleural irritation from chest drains (13). Open thoracic surgery is characterized by the need to artificially disconnect the latissimus dorsi muscle and brace the rib cage; thus, limiting shoulder movement and causing ISP (13). Two of the more recognized causes are phrenic nerve pain and shoulder ligament strain (12). There are two main components with regard to the treatment of ISP, nerve blocks and drug therapy, and there are many treatments for nerve infiltration, including phrenic nerve infiltration (14–17), suprascapular nerve block (SNB) (9, 18), transcutaneous electrical nerve stimulation (19), spinal cord stimulation (20) subcutaneous targeted neuromodulation technique (21), low-volume interscalene brachial plexus block (22), brachial plexus block (23), and ipsilateral stellate ganglion block (24). A randomized double-blind comparative study of phrenic nerve infiltration and SNB for ISP after thoracic surgery confirmed the effectiveness of this treatment option (5, 16). However, drug therapy involves the application of nonsteroidal anti-inflammatory drugs or opioid medications for symptomatic pain management and this therapy not targeted (25–28).

The characteristics of ISP caused by open thoracic surgery and single-operating port thoracoscopic surgery are different because conventional open thoracic surgery requires disconnection of the dorsal muscle groups and bracing of the ribs, which has a higher incidence of postoperative ISP, whereas single-port or single-operating port thoracoscopic surgery does not cause such an injury. We believe that a possible reason for the occurrence of ISP after single-operator port thoracoscopic surgery is that thoracic surgery requires the patient to remain in a lateral position throughout the procedure and the upper arm needs to be kept in a forward-flexed position throughout the procedure; the prolonged passive position can cause postoperative strain on the patient's shoulder muscles and ligaments, which can lead to postoperative ISP (6). Although there are some relevant studies to guide the treatment of ISP, how to avoid this medically induced cause of decreased quality of life in patients has not been adequately studied.

Therefore, we conducted this single-center observational study with the aim of observing the risk factors associated with the occurrence of ISP after single-operator port thoracoscopic surgery. These included the effects, such as age, sex, duration of surgery, and height on postoperative ISP.

## Methods

A total of 140 patients treated in the Department of Lung Cancer Surgery, Tianjin Medical University General Hospital between July 6, 2020 and August 21, 2020 were included in this study. The inclusion criteria were as follows: (1) Aged < 80 years and >18 years; (2) single-operating port thoracoscopic procedures in the lateral position; and (3) patients are able to cooperate in completing all movements required by the Constant-Murley shoulder function scoring system. The Constatn-Marley scale presents a method of assessment by way of a simple 100-point scoring system incorporating subjective measures of pain and activity and objective tests of range of movement and power. Inter-observer error of the scoring system was low. It is a valid assessment tool used in clinical practice all over the world. In addition, it is very sensitive in picking up even small changes in shoulder function. It can be used for any pathology affecting the shoulder (29). The exclusion criteria were as follows: (1) single-operator port thoracoscopy with intermediate open thoracic or secondary surgery; (2) any previous disease of the upper extremity or shoulder joint that could cause pain or sensory abnormalities in the upper extremity or impaired shoulder motion; (3) patients with mental illness, communication disorders, or language differences; (4) patients who refused to participate in the trial or refused follow-up. The study was conducted with the approval of the Ethics Committee of the General Hospital of Tianjin Medical University (Ethical NO. IRB2021-WZ-055), and all the patients signed the informed consent form.

**Figure 1:**
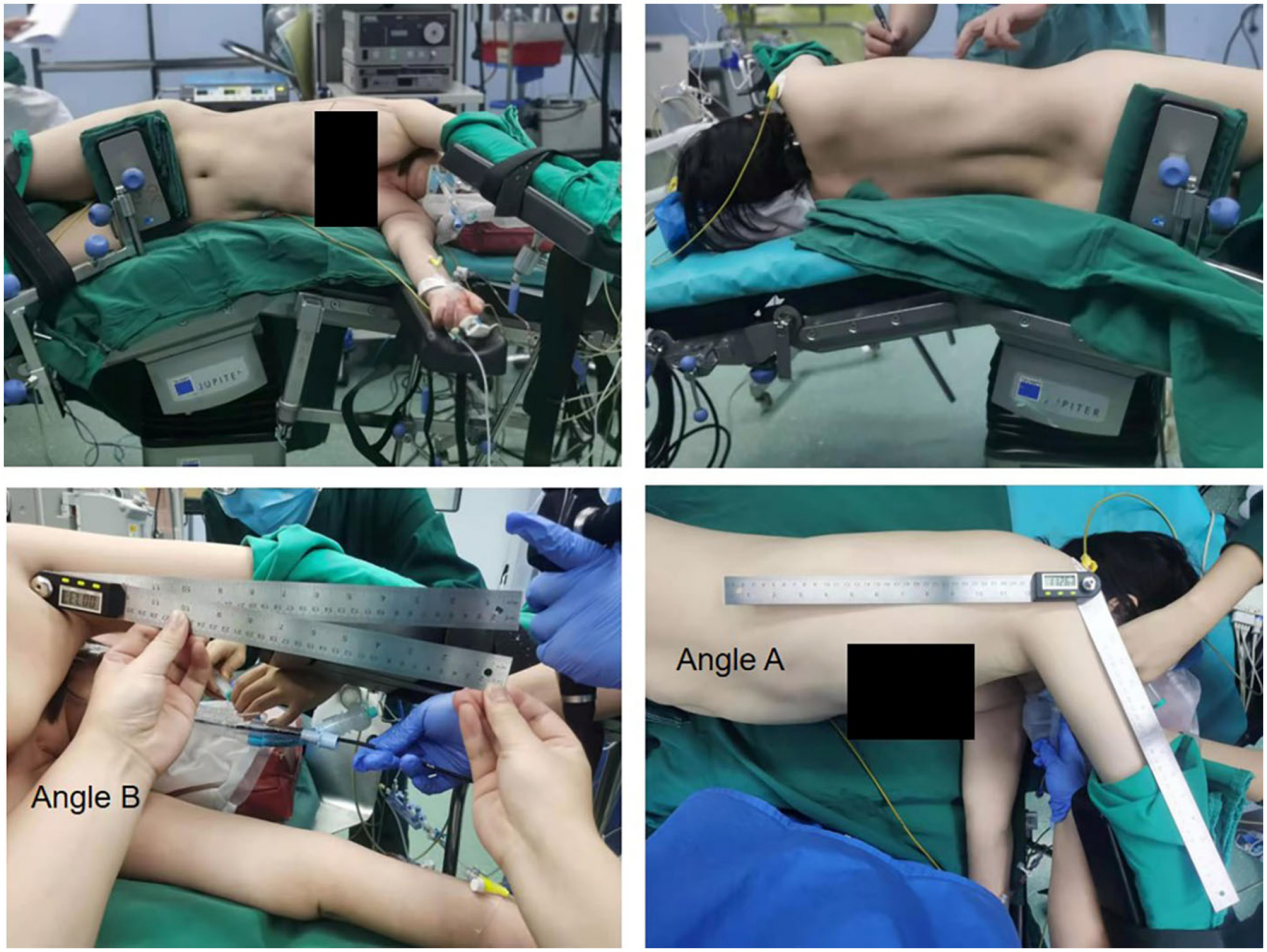
The representative position of patients during the surgery and measurement mode of angle A and B.

On the day of the patient's surgery, the upper arm angle was measured by an experienced observation recorder after placement in the preoperative position. The shoulder flexion was chosen according to the condition of patients' body and comfort-driven position of the surgeon during operation. We defined angle A as the flexion angle of operative shoulder joint, angle B was the adduction angle of operative shoulder joint, as shown in Figure 1. In addition, in terms of angle B, we defined the angle of 0° when the patient's upper arm was abducted to be parallel to the coronal position for better statistical data. Patients were followed up at 3 days, 2 weeks, and 6 weeks postoperatively, and scores were completed according to the Constant-Murley shoulder function rating scale.

## Results

A total of 140 patients were included in this study, of whom 57 (40.7%) were men and 83 (59.3%) were women. The maximum and minimum age of these patients was 79 years and 20 years, respectively; the maximum and minimum height was 188 and 145 cm, respectively. Significant subjective pain occurred in 13 patients (9%) after single-operating port thoracoscopy (Figure 1; Table 1). The range of Angle A and Angle B was 95–135° and 78–110°, respectively (Table 1). The postoperative 3-day, 2-week, and 6-week Constant-Murley shoulder function rating scale scores with maximum values of 85.46, 85.46, and 85.46, respectively; minimum values of 65.08, 73.10, and 77.44, respectively; and median values of 79.44, 82.78, and 82.78, respectively, are shown in Table 2.

**Table 1:** Descriptive statistics for the study population.

**Variables**	**Description**	**Day 3**		**Day 14**				**Day 42**			
		**OR (95% CI)** ***P***		**Univariate logistic** **regression analysis**		**Multivariate logistic** **regression analysis**		**Univariate logistic** **regression analysis**		**Multivariate logistic** **regression analysis**	
				**OR (95% CI)** ***P***		**OR (95% CI)** ***P***		**OR (95% CI)** ***P***		**OR (95% CI)** ***P***	
Male/Female	57/83	0.515 [0.396, 1.591]	0.515	1.716 [0.839, 3.509]	0.139			1.64 [0.819, 3.282]	0.163		
Age (Median, IQR)	61(54-68)	1.094 [1.046, 1.143]	<0.001	1.087 [1.041,1.134]	<0.001	1.098 [1.046, 1.152]	<0.001	1.08 [1.034, 1.118]	<0.001	1.079 [1.036, 1.124]	<0.001
Height (cm)(Median, IQR)	165(160-170)	0.965 [0.921, 1.011]	0.132	0.931 [0.886, 0.979]	0.005	0.923 [0.871, 0.977]	0.006	0.94[0.895, 0.984]	0.009	0.933 [0.886, 0.983]	0.009
Weight (Kg)(SD)	66.53 ± 11.58	0.988 [0.959, 1.018]	0.430	0.987 [0.957, 1.017]	0.375			0.98 [0.947, 1.006]	0.111		
BMI(Kg/m2)(SD)	24.29 ± 3.20	1.003 [0.901, 1.117]	0.959	1.054 [0.946, 1.175]	0.340			0.99 [0.890, 1.098]	0.828		
Duration of surgery (min)(Median,IQR)	135(90-180)	1.002 [0.996, 1.007]	0.586	0.998 [0.992, 1.004]	0.515			1.00[0.993, 1.005]	0.727		
Angle A(°)(Median, IQR)	108(105-116)	1.029 [0.984, 1.075]	0.214	1.062 [1.014, 1.112]	0.011	1.064 [1.011, 1.121]	0.018	1.04 [0.996, 1.088]	0.076		
Angle B(°)(Median, IQR)	87(84-93)	1.002 [0.948, 1.059]	0.946	0.991 [0.937, 1.048]	0.741			0.98 [0.925, 1.034]	0.433		

*SD, standard deviation; OR, odds ratio; P, Value*.

**Table 2:** Descriptive statistics for the ISP population.

**Time**	**N**	**Min**	**Max**	**Mean value**	**SD**
3 days	13	65.08	73.1	69.4569	2.69875
2 weeks	13	73.1	80.78	77.6708	2.15486
6 weeks	13	77.44	80.78	78.1746	1.35172

*N, Number; Min, Minimum; Max, Maximum; SD, Standard deviation*.

### ISP in Relation to Time

**Figure 2:**
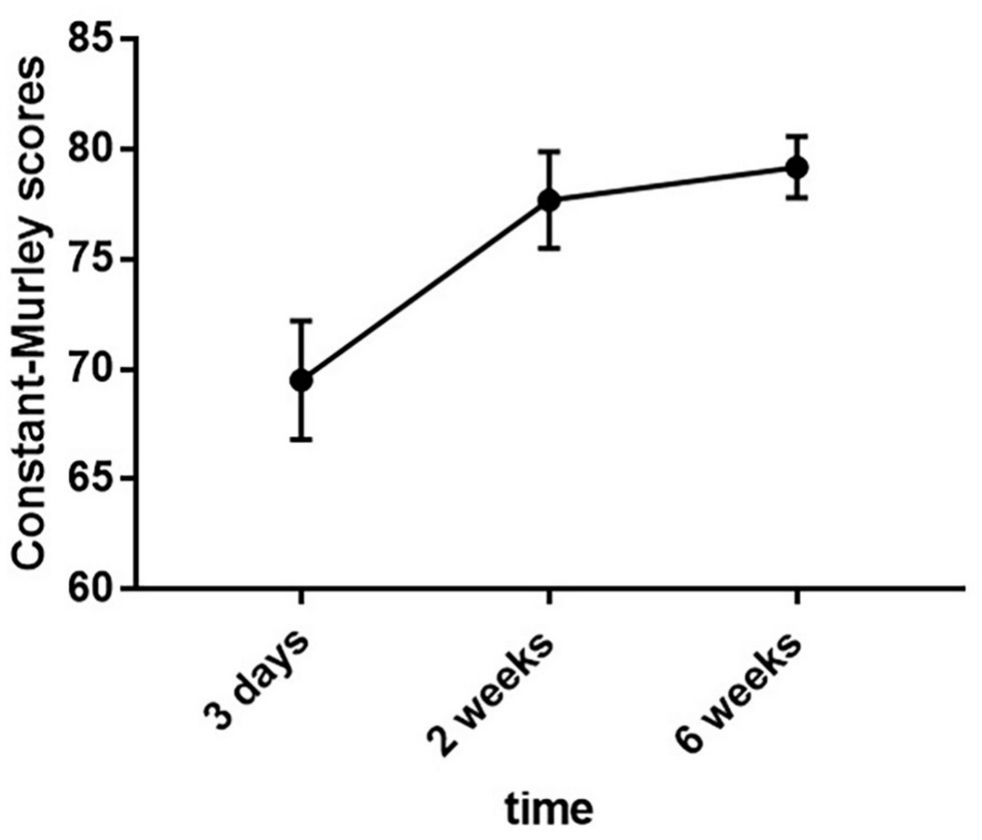
Trends in scores for the ISP patient at three postoperative time points.

Patients had the lowest shoulder function scores up to 3 days postoperatively, which largely stabilized after 2 weeks. A total of 13 patients with resting subjective pain after surgery had mean and standard deviation of shoulder function scores of 69.5 ± 2.7, 77.7 ± 2.2, and 79.2 ± 1.4 at 3 days, 2 weeks, and 6 weeks postoperatively, respectively. It can be seen that the postoperative scores gradually increased with time, and the trend of increasing scores from 3 days to 2 weeks postoperatively was more obvious (Figure 2; Table 2).

### Analysis of Risk Factors Associated With ISP

A univariate logistic regression analysis of the data at postoperative day 3 showed that age was an independent risk factor for low shoulder function scores at 3 days postoperatively (OR: 1.094 [1.046–1.143]; *P* < 0.001).

The results of univariate logistic regression analysis at 2 weeks postoperatively showed that age, height, and Angle A were independent influencing factors on low shoulder function scores (OR: 1.087 [1.041–1.134], 0.931 [0.886–0.979], and 1.062 [1.014–1.112], respectively). The three variables that were statistically significant in the univariate logistic regression were included in the multivariate logistic regression model, and the results showed that age and Angle A were independent risk factors for low shoulder function scores at 2 weeks postoperatively (OR: 1.098 [1.046–1.152] and 1.064 [1.011–1.121], respectively), while height was an independent protective factor (OR: 0.923 [0.871–0.977]), all with statistically significant *P*-values.

The univariate analysis showed that age and height were both influencing factors for 6-week postoperative scores (OR: 1.08 [1.034–1.118], *P* < 0.001 and 0.94 [0.895–0.984], *P* = 0.009, respectively), with statistically significant *P*-values. The two variables with significance in the univariate logistic regression were included in a multivariate logistic regression model, and the results showed that age was an independent risk factor for low shoulder function scores at 2 weeks postoperatively (OR: 0.94 [0.895–0.984]) and height was an independent protective factor for low shoulder function scores at 6 weeks postoperatively (OR: 0.933 [0.886–0.983], *P* = 0.009), both with statistically significant *P*-values.

As seen in Table 1, no statistically significant relationship between Angle B and ISP, with *P*-values of 0.946, 0.741, and 0.433 was observed. Angle B was not an independent risk or protective factor for postoperative shoulder function scores.

### ISP-Related Angle Subgroup Analysis

Angle A was an independent risk factor for reduced shoulder function scores at 2 weeks postoperatively (Table 1), using the Jorden index formula: Jorden index = sensitivity + specificity – 1. The cutoff value is the largest value of the Jorden index, i.e., 113°.

**Figure 3:**
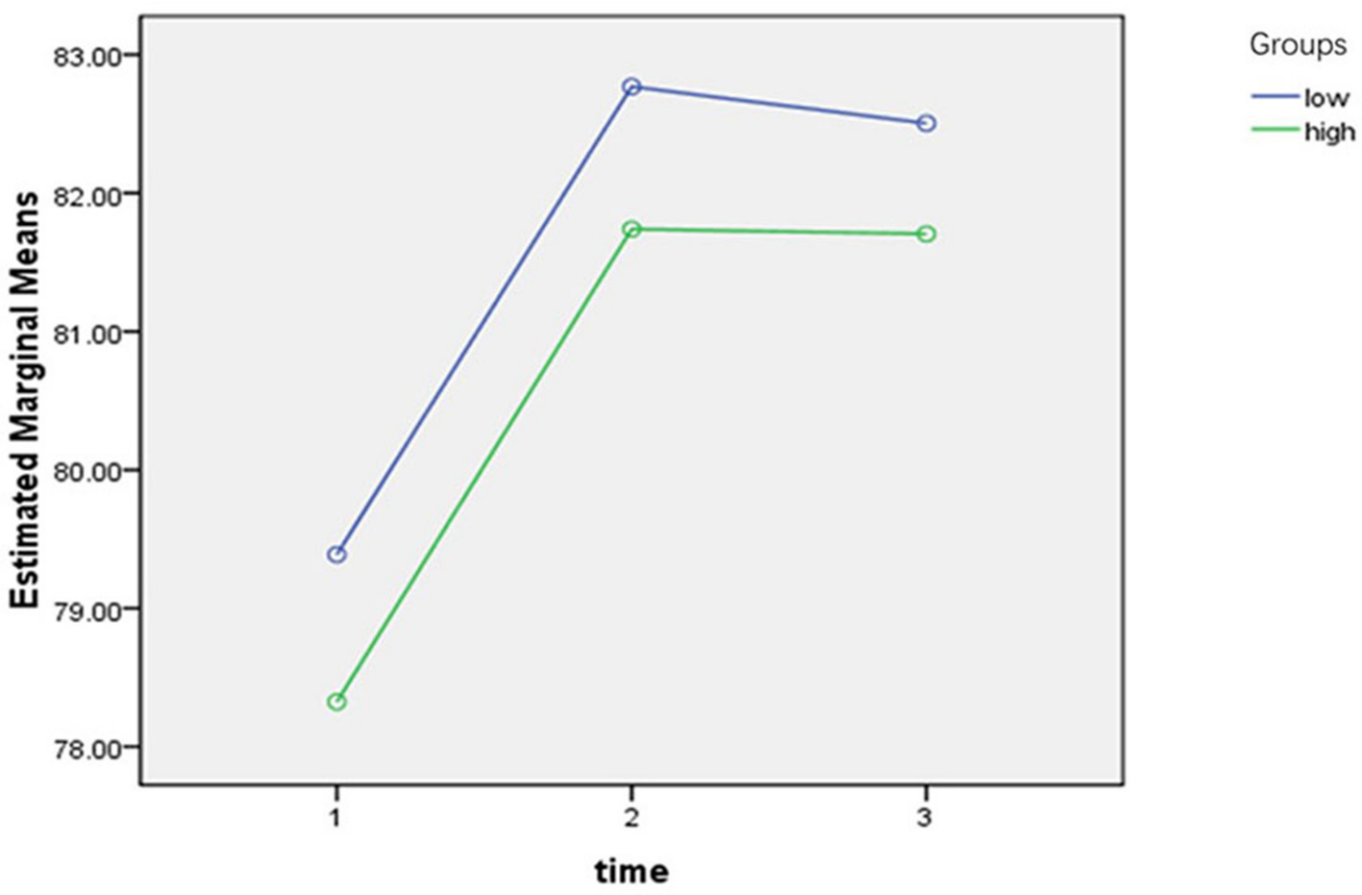
Angle A and repeated measures analysis of variance at three postoperative time points.

To demonstrate a correlation between Angle A and low scores, we used 113° as the cutoff, defined >113° as the high-risk group and <113° as the low-risk group, and performed a repeated measures analysis of variance (ANOVA) on the scores at each of the three postoperative time points. The trend in score change over time was statistically significant, with no interaction between temporal change in score and Angle A grouping, and statistically significant differences in the trend in score change between groups at the three postoperative time points according to Angle A grouping (Figure 3; Tables 3, 4). The results indicated that the group with a greater Angle A value had lower scores at all three postoperative time points.

**Table 3:** Comparison of baseline characteristics between the high- and low-risk groups in Angle A.

**Variables**		**high-risk group**	**low-risk group**	***P***
		**(≥113**°**)**	**(<113**°**)**	
		***N* = 49**	***N* = 91**	
Sex	Male	18(36.7%)	39(42.9%)	0.482
	Female	31(63.3%)	52(57.1%)	
Age(year)(Median,IQR)		61(56,69)	60(52,67)	0.317
Height(cm) (Median,IQR)		163(158,170)	165(160,170)	0.142
Duration of surgery (min)(Median,IQR)		130(93,183)	140(90,180)	0.922
Weight(Kg)(SD)		67.54 ± 10.86	65.99 ± 11.98	0.452
BMI(Kg/m2)(SD)		25.08 ± 2.92	23.86 ± 3.28	0.030

*BMI, Body Mass Index; SD, Standard deviation; Kg, Kilogram*.

**Table 4:** Trends in scores for the high and low-risk groups of Angle A at three postoperative time points.

	**Df**	**F**	***P***
Angle A	1	5.118	0.025
time	1.441	176.348	<0.001
Angle A time	1.441	0.248	0.706

After dividing Angle A into high- and low-risk groups with a cutoff point of 113°, the relationship between Angle A and each variable was compared. The differences in sex, age, height, weight, and operation time were not statistically significant between the two groups. Body mass index (BMI) was statistically significant between the two groups and BMI was greater in the high-risk group with a *P*-value of 0.03 (Table 3).

## Discussion

ISP is a common postoperative complication after thoracic surgery, with an incidence of 42-85% (11, 30). To date, the specific etiology of ISP remains unknown; therefore, management of ISP is challenging. A longitudinal observational prospective cohort study found a significant correlation between postoperative muscle dysfunction of shoulder skeletal muscle and VATS, and this dysfunction still existed at 1 month postoperatively (5). Previous studies related to ISP have focused on open thoracic surgery (11), and explanations for the cause of pain have mostly been limited to surgical trauma, such as surgically induced nerve pull pain (3–5), muscle damage, and postoperative chest drain irritation (13); hence, the corresponding management measures for ISP are symptomatic analgesic treatments, such as nerve blocks (14–17) and the application of analgesics (25–28). A randomized double-blind study of phrenic and SNB on the occurrence of ISP after thoracic surgery showed a significant reduction in shoulder pain in the group by the application of 2% lidocaine for phrenic nerve block (5). However, non-surgical trauma factors associated with ISP have been rarely reported; therefore, the main objective of this study was to investigate the non-surgical trauma influences on postoperative shoulder function impairment caused by single-operator port thoracoscopic surgery, with a focus on the correlation between intraoperative upper extremity positioning angle and shoulder function impairment, to reduce medically induced injuries beyond surgical trauma from the perspective of operating room management.

Previous studies have focused on ISP owning to nerve pain, and the most commonly used method is pain scoring, i.e., the visual reference scale (VRS) and visual analog scale (VAS) (5), which is simple and easy to operate. However, because of the wide age distribution of surgical patients, the tolerance of postoperative pain varies significantly among different age groups, and the VRS and VAS scores are highly subjective, which can easily interfere with the results. In addition, ISP causes pain while adversely affecting patients' shoulder mobility and quality of life. Hence, the Constant-Murley shoulder function scale, which is specifically designed to diagnostically assess shoulder function, was chosen for this study to quantify the degree to which the shoulder joint was affected by pain, mobility, and psychological factors and to minimize the bias caused by differences in pain tolerance on statistical results. This scale minimized the bias of the results. This was used to comprehensively assess whether the intraoperative position of the upper arm could cause physical damage to the shoulder joint. Shoulder pain has been reported in 20% of patients in the postoperative period (7). It has been reported that shoulder pain levels were highest on postoperative day 1 and gradually decreased over time. In contrast, our study found that the high incidence of postoperative upper extremity pain was at postoperative day 3, and ~9% of patients undergoing single-operating port thoracoscopic surgery experienced varying degrees of ISP at rest after surgery, and even without subjective pain, and some patients exhibited mild limitation of shoulder range of motion, similar to data reported in the literature (8). Pain mostly resolved at 2 weeks postoperatively, and at 6 weeks postoperatively subjective pain disappeared, and most remained with mild limitation of function.

The VATS has the advantages of small surgical incisions and short operation time. This approach is beneficial in reducing incisional pain during the early postoperative period (31, 32). Several previous studies have reported mechanisms of ISP, including severe bronchial injury, muscle strain owning to shoulder immobilization, and pain caused by the mediastinal and diaphragmatic pleura innervated by the phrenic nerve (5, 30). However, we found that the effect of intraoperative upper limb angulation on postoperative shoulder pain has not been studied. The results of this study showed that the patients' angle of adduction and abduction (Angle B) was not related to the risk of postoperative ISP, indicating that the angle of adduction or abduction has a small effect on patient's shoulder function and does not increase the risk of postoperative ISP in normal clinical work. However, in this study we observed that the patients' angle of anterior flexion of the upper extremity (Angle A) increased the risk of postoperative ISP in patients and this was limited to the time point of 2 weeks postoperatively, while for 3 days or 6 weeks postoperatively, no risk of ISP was observed for patients with increased Angle A. To further explore whether Angle A affects postoperative shoulder scores, we divided Angle A into high- and low-risk groups and performed a repeated measures ANOVA on the scores at three postoperative time points. We found that the scores in the group with a higher Angle A value were relatively low at all three postoperative time points, and this variation trend in scores was statistically significant. Therefore, we concluded that excessive Angle A would cause functional impairment of the shoulder joint.

In the preoperative positioning, the range of motion of Angle B was relatively small and basically in the normal range of motion of the shoulder joint in this plane; hence, no correlation between Angle B and postoperative ISP was observed. There is a certain amount of tension in the shoulder joint when Angle A is extremely large, and in cases where the shoulder joint has poor tolerance owning to other reasons, such as the patient's old age, the prolonged passive position during surgery can cause pain and movement disorder of the shoulder joint. As this degree of injury is generally the result of muscle ligament strain around the shoulder joint, most cases do not have organic lesions and this injury resolves over time in postoperative rehabilitation; most patients' shoulder discomfort is relieved at 6 weeks postoperatively. Hence, no statistically significant effect of Angle A on shoulder scores was observed. To reduce the occurrence of postoperative shoulder pain caused by Angle A in clinical work, we tried to identify the critical value of Angle A as a reference; the patient's postoperative shoulder score was significantly reduced when Angle A was >113°; hence, the patient's upper arm forward flexion angle should not exceed 113°.

This study also found that age was the main risk factor for the occurrence of ISP. Age was the only variable among all indicators that correlated with shoulder function scores at all the three postoperative time periods, indicating that age is an independent risk factor for causing postoperative shoulder function impairment after single-operator port thoracoscopy, and the older the age, the greater the likelihood of postoperative shoulder function impairment. However, height was a protective factor for shoulder function impairment at 2 and 6 weeks postoperatively, and the taller the height, the lesser was the probability of postoperative shoulder function impairment. During preoperative positioning, the staff would adjust the patient's upper arm forward flexion angle appropriately to increase the comfort of intraoperative operation. Tall and thin patients would provide a wider operating space for the surgeon, while for short and fat patients because of the narrow operating space; the surgeon might complete the operation by increasing the patient's upper arm forward flexion angle. It is clear from the above findings that increasing the patient's upper arm forward flexion angle increases the patient's risk of developing ISP postoperatively. In addition, the results of this study revealed a correlation between Angle A and BMI, the greater the angle, the greater the BMI, which also confirms the above view.

However, no cases of chronic ISP were collected in this study, which might be owning to the low incidence of chronic ISP and the fact that the number of cases in this study was small; thus, further multicenter studies with a large sample size are warranted.

## Conclusion

ISP was closely related to the angle of anterior flexion of the upper arm on the patient's operative side intraoperatively. The increase in the degree of postoperative shoulder injury is more pronounced for an anterior flexion angle of >113°. Therefore, we recommend that the angle of anterior flexion of the upper extremity should be <113° intraoperatively.

## Data Availability Statement

The original contributions presented in the study are included in the article/supplementary material, further inquiries can be directed to the corresponding author/s.

## Ethics Statement

This study was conducted with the approval of the Ethics Committee of the General Hospital of Tianjin Medical University (Ethical No. IRB2021-WZ-055), and all the patients signed the informed consent form. The patients/participants provided their written informed consent to participate in this study. Written informed consent was obtained from the individual(s) for the publication of any potentially identifiable images or data included in this article.

## Author Contributions

DR: conceptualization, patient's selection, data analysis, project administration, writing—original draft, and writing—review and editing. BZ: conceptualization, data analysis, and writing—original draft. JX: conceptualization, patient's selection, and writing—original draft. RL: patient's selection and writing—original draft. JW: data selection and analysis. HH: patient's selection and data analysis. HZ, JZ, and HW: data analysis. XX: conceptualization and supervision. ML: data selection and conceptualization. SX: conceptualization, writing—review and editing, and supervision. ZS: conceptualization and supervision.

## Funding

This study was funded by the National Natural Science Foundation of China (No. 82172776), the Tianjin Key Project of Natural Science Foundation (No. 17JCZDJC36200), Tianjin Science and Technology Plan Project (19ZXDBSY00060), and Tianjin Science and Technology Plan Project (2013KZ121).

## Conflict of Interest

The authors declare that the research was conducted in the absence of any commercial or financial relationships that could be construed as a potential conflict of interest.

## Publisher's Note

All claims expressed in this article are solely those of the authors and do not necessarily represent those of their affiliated organizations, or those of the publisher, the editors and the reviewers. Any product that may be evaluated in this article, or claim that may be made by its manufacturer, is not guaranteed or endorsed by the publisher.
